# M-Calpain Activation Facilitates Seizure Induced KCC2 Down Regulation

**DOI:** 10.3389/fnmol.2018.00287

**Published:** 2018-08-21

**Authors:** Li Wan, Liang Ren, Lulan Chen, Guoxiang Wang, Xu Liu, Benjamin H. Wang, Yun Wang

**Affiliations:** Department of Neurology, Institutes of Brain Science & State Key Laboratory of Medical Neurobiology, Zhongshan Hospital, Fudan University, Shanghai, China

**Keywords:** epilepsy, calpain, KCC2, MAPK/ERK, PTZ

## Abstract

Potassium chloride co-transporter 2 (KCC2), a major chloride transporter that maintains GABA_A_ receptor inhibition in mature mammalian neurons, is down-regulated in the hippocampus during epileptogenesis. Impaired KCC2 function accelerates or facilitates seizure onset. Calpain, with two main subtypes of m- and μ-calpain, is a Ca^2+^-dependent cysteine protease that mediates the nonlysosomal degradation of KCC2. Although recent studies have demonstrated that calpain inhibitors exert antiepileptic and neuroprotective effects in animal models of acute and chronic epilepsy, whether calpain activation affects seizure induction through KCC2 degradation remains unknown. Our results showed that: (1) Blockade of calpain by non-selective calpain inhibitor MDL-28170 prevented convulsant stimulation induced KCC2 downregulation, and reduced the incidence and the severity of pentylenetetrazole (PTZ) induced seizures. (2) m-calpain, but not μ-calpain, inhibitor mimicked MDL-28170 effect on preventing KCC2 downregulation. (3) Phosphorylation of m-calpain has been significantly enhanced during seizure onset, which was partly mediated by the calcium independent MAPK/ERK signaling pathway activation. (4) MAPK/ERK signaling blockade also had similar effect as total calpain blockade on both KCC2 downregulation and animal seizure induction. The results indicate that upregulated m-calpain activation by MAPK/ERK during convulsant stimulation down regulates both cytoplasm- and membrane KCC2, and in turn facilitates seizure induction. This finding may provide a foundation for the development of highly effective antiepileptic drugs targeting of m-calpain.

## Background

The inhibitory effect of GABA_A_ receptors has an important role in the maintenance of normal brain function and protection against epileptogenesis. These effects rely on a low intracellular Cl^−^ ([Cl^−^]_i_) and high extracellular Cl^−^ transmembrane gradient, which is established through Cl^−^ transport by potassium chloride co-transporter 2 (KCC2) in mature neurons (Chamma et al., 2012; Mahadevan and Woodin, 2016; Wu et al., 2016). KCC2, the only KCC family member distributed in the central nervous system, mainly pumps Cl^−^ out of neurons and maintains low [Cl^−^]_i_ (Kahle et al., 2008; Loscher et al., 2013). Numerous studies have shown that epileptic seizure stimuli significantly down-regulate KCC2 expression. KCC2 down-regulation weakens KCC2 function and GABA inhibition, in turn, accelerates and facilitates seizure induction (Munakata et al., 2007; Eftekhari et al., 2014; Hübner, 2014; Kahle et al., 2014, 2016; Karlócai et al., 2016; Chen et al., 2017). However, the mechanism of seizure-induced KCC2 down-regulation remains inconclusive despite the abundance of studies on seizure development (Li et al., 2008; Puskarjov et al., 2015; González, 2016).

Calpain is a Ca^2+^-dependent cysteine protease that is ubiquitously expressed in mammals (Zimmerman and Schlaepfer, 1984; Ono et al., 2016). Despite its ubiquity and early discovery, calpain remains enigmatic. Two major isoforms of calpain exist, of which μ-calpain is activated by 3–50 μM Ca^2+^ and m-calpain is activated by 400–800 μM Ca^2+^ (Liu et al., 2008; Sorimachi et al., 2011a; Zanardelli et al., 2013), and both of them exist in mature neurons in central nervous system (Goll et al., 2003). However, in a specific neuron, the distribution of μ-calpain and m-calpain is considered to have regional specificity. The morphological evidence shows that μ-calpain mostly distributed at the synapse region, which facilitates its regulation on synaptic function through the effect to the cytoskeleton, the scaffold protein and the glutamate receptor (Liu et al., 2008). However, the ultrastructural localization of m-calpain in neurons is still unclear. Functional studies have shown that, in excitatory neurons, activation of synaptic NMDA receptors causes the activation of the μ-calpain (Xu et al., 2009), while the extra-synaptic NMDA receptor specifically activates m-calpain (Chen et al., 2007). Calpain activation has been reported to involve in many neurological disorders, such as traumatic brain injury (TBI), Alzheimer disease (AD), epilepsy, etc. (Sierra-Paredes et al., 1999; Araujo et al., 2008; Li et al., 2013; Lam et al., 2017; Nam et al., 2017). In terms of seizure, it is found that calpain was over-activated in KA induced seizure rats, and this effect could be blocked by calpain inhibitor MDL-28170 (Araujo et al., 2008). In another study in pilocarpine-induced status epilepticus (SE) model, calpain was also found to be abnormally activated, and MDL-28170 was proved to ameliorates seizure burden in that case (Lam et al., 2017). However, the specific mechanism underlying these effects remains unclear. One of the underlying mechanisms of calpain involvement in epilepsy might attribute to its regulation of KCC2 degradation, since KCC2 down regulation has been directly associated to seizure induction (Chen et al., 2017). KCC2 may follow two routes in clathrin-mediated KCC2 endocytosis. One route involves vesicle recycling, wherein KCC2 is reassembled and resumes its function in the plasma membrane. The other route involves degradation via a chain reaction mediated by calpain. Thus, the over-activation of calpain can diminish the intracellular KCC2 pool, eventually decreasing the expression of KCC2 on plasma membrane (Puskarjov et al., 2012, 2015; Zhou et al., 2012; Chamma et al., 2013). These effects imply that calpain participates in epileptogenesis by regulating KCC2.

Meanwhile, calpain itself is subject to complex regulation. Recent studies have shown that in addition to regulation by intracellular Ca^2+^ concentration ([Ca^2+^]_i_), m-calpain activity is also regulated through MAPK/ERK-mediated phosphorylation (Chen et al., 2010; Liu et al., 2016). In Glading et al. (2004) provided a detailed discussion of MAKP/ERK-mediated m-calpain phosphorylation under physiological conditions and identified ser50 as the regulatory site of m-calpain. However, whether MAPK/ERK-mediated phosphorylation of m-calpain involves in seizure induction is still unknown.

Recent reports have shown that the contradictory effects exerted by nonselective calpain inhibitors may be attributed to the different or opposing roles of μ-calpain and m-calpain under certain pathological conditions (Baudry and Bi, 2016; Wang et al., 2016; Yin et al., 2016). Here, we report that blockade of calpain by calpain inhibitor MDL-28170 suppressed the seizure induction, as well as the KCC2 downregulation. We further demonstrated that m-calpain, but not μ-calpain, phosphorylation, which was partly mediated by the calcium independent MAPK/ERK signaling pathway activation, regulated KCC2 down regulation and hence the seizure onset.

## Materials and Methods

### Ethics Statement

All the animal experiments were approved by the Local Committees of the Use of the Laboratory Animals, Fudan University (Shanghai, China) and were carried out in accordance with the guidelines and regulations of National Natural Science Foundation of China animal research. In addition, institutional safety and biosecurity procedures were followed according to “Laboratories-General requirements for biosafety (GB 19489-2008).”

### Animal Preparation

Male SD rats were purchased from Shanghai Slack Experimental Animals Inc. The weight of the animal is 180–220 g, aged 5–6 weeks. Rats were housed in a regulated environment (22 ± 1°C) with a 12 h light–dark cycle, and food and water were available *ad libitum*.

### Hippocampal Slices Preparation

Rats were anesthetized by intraperitoneal injection of 1.25% pentobarbital sodium at a dose of 0.1 ml per 100 g weight. After being fully anesthetized, the rats were decapitated and their brains were separated and cooled in iced ACSF (in mmol/L: NaCl 124, KCl 3.3, KH_2_PO_4_ 1.2, NaHCO_3_ 26, CaCl_2_ 2.5, MgSO_4_ 2.4, Glucose 10) for 1 min, then trimmed on ice to expose the hippocampus as much as possible. The brain was then fixed on cryogenic vibration microtome for slicing. The brain was bathed in iced ACSF throughout the slicing. For immunoblotting the slice thickness was 350 μm and for immunohistochemistry the thickness was 100 μm. Slices were then transferred to an interface tissue chamber and superfused with ACSF at a temperature of 31–32°C for 30 min. After that the slices were maintained at room temperature (RT) for 1 h to recover function. By then the slices were ready for further pharmacological treatments and sampling.

### Hippocampal Slices Perfusion

Fresh hippocampal slices prepared from above were randomly divided into different groups at same numbers. Slices from one single preparation were seen as the same batch. Since our previous study, Chen et al. (2017) has reported that plasma membrane KCC2 (mKCC2) down-regulation in 0 Mg^2+^ induced *in vitro* seizure model reached maximal at 2 h after 0 Mg^2+^ ACSF incubation, we chose 2 h as the time scale of 0 Mg^2+^ treatment in our current study. The time scale of BDNF treatment (#450-02, PeproTech, 200 ng/ml) was also set to 2 h according to a previous research (Rivera et al., 2002). In corresponding vehicle control or intervention groups, slices were also treated at same time with one of the following agents: MDL-28170: #ab145601, abcam, 50 μM; PD98059: #ab120234, abcam, 25 μM ; K252a: #420298, Calbiochem, 200 nM; Tautomycetin: #2305, Tocris, 20 nM; BAPTA-AM: # A1076, Sigma, 10 μM; Calpain Inhibitor I: # A6185-5MG, Sigma, 100 μM; Calpain Inhibitor IV: # 208724, Calbiochem, 200 μM. All drugs were dissolved in DMSO before being added to ACSF. The final concentration of DMSO was 0.1% in each treatment and bath incubation.

### EEG Recording and Behavior Assays

Due to its low neurotoxicity and stability in inducing seizure, pentylenetetrazole- (PTZ, 50 mg/kg) induced seizure model was chosen in this study. Although PTZ has been widely accepted as a GABA_A_ receptor antagonist, its actual mechanism in inducing seizure in *in vivo* animal model is not fully defined, since PTZ has been also reported to blockade of certain ion channels (Papp et al., 1987) and *in vitro* application of PTZ on hippocampal slices failed to evoke epileptiform burst activities as other GABA_A_ receptor antagonist do (unpublished data). Behavioral seizures in freely moving rat combination with electroencephalograph (EEG) were recorded as described previously (Kong et al., 2010). In generally, male SD rat (180–220 g) were anesthetized with sodium pentobarbital (60 mg/kg, i.p.) and mounted in a stereotaxic apparatus with body temperature maintained at 37°C. Two stainless steel screws (1 mm in diameter) were inserted through the skull with one screw serving as recording electrode above the hippocampus (AP −3.8 mm and ML 2.0 mm) and the other as reference electrode above the forehead. Screws were then connected to a connector-plug with wires for later connecting to recording leads. All electrodes were attached to a micro-connector and fixed onto the skull with dental cement. After surgery, animals were allowed to recover for at least 5 days before the experiments. For experiment, rats were transferred to a plexiglas cage (25 × 25 × 40 cm) and habituated therein for at least 30 min, before intraperitoneal injection with either MDL-28170 (#ab145601, abcam, 50 mg/kg) or SL-327 (#HY-15437, MCE, 50 mg/kg) or equal volume vehicle (DMSO) in different groups as pre-treatment. Thirty minutes after that, PTZ (50 mg/kg) was injected intraperitoneally to induce seizure. Epileptic behavior and EEG were simultaneously recorded for 1 h after PTZ kindling, and then terminated by intraperitoneal injection of pentobarbital.

The EEG signals were sampled at rate of 2,500 Hz, analog inputs were amplified (1,000 times) and filtered (0.3–1 kHz) by using a NeuroLog System (Digitimer Ltd., Hearts, UK) and digitized with CED Micro 1401 (Cambridge Electronic Design, Cambridge, UK) and recorded in a personal computer using Spike two software (version 6.0, Cambridge Electronic Design, Cambridge, UK). Each recording lasted at least 1 h after PTZ injection. Classic Racine classification method was introduced to scale the PTZ-induced seizure severity: R1: chewing, blinking, facial or beard trembling twitching, stare, daze; R2: nodding, repeated scratch, circle around and wet dog shakes (WDS); R3: unilateral forelimb clonus, tail-erecting and back arching; R4: rearing with bilateral forelimb clonus; R5: rearing and falling (loss of postural control).

### Immunostaining

Hippocampal slices (100 μm) from different intervention group were fixed by 4% paraformaldehyde (PFA, Sigma) for 30 min then rinses in TBS. After that the slices were set in 0.2% Triton X-100 (Sigma) and 10% normal donkey serum (NDS, Millipore) in TBS for permeabilize and blocking at RT for 2 h. Then slices were incubated at 4°C overnight with primary antibody (rabbit anti-KCC2, #07-432, Millipore, 1:300; Rabbit anti-NeuN, #24307, CST, 1:400) diluted in 10% NDS. After rinses, slices were incubated with corresponding secondary antibodies (donkey anti-rabbit conjugated to Alexa Fluor 594 or 488, 1:300; Molecular Probe) diluted in 10% NDS at RT for 2 h. After rinsed, the slices were mounted on slides and cover-slipped with ProLong Gold antifade reagent (Molecular Probe). Images were acquired with confocal scanning microscope with 25× objective (A1 R, Nikon).

### Plasma Membrane and Cytoplasm Protein Extraction and Sample Preparation

The plasma membrane and cytoplasm protein fractions were prepared followed by standard procedures provided by the Membrane Protein Extraction Kit purchased from Biovision (#K268-50, containing Homogenize Buffer, Protease Inhibitor Cocktail, Upper Phase Solution, and Lower Phase Solution). Hippocampal slices were dissociated to preserve only the hippocampus part under a dissecting microscope on ice, and then quickly homogenized in pre-cooled Homogenize Buffer containing 1/500 Protease Inhibitor Cocktail. The homogenate was centrifuged at 700× *g* for 10 min at 4°C. Then the supernatant was transferred to a new vial and centrifuged at 10,000× *g* for 30 min at 4°C. Collect both the pellet (the total membrane proteins) and the supernatant (the cytoplasm fraction). The total membrane proteins were further purified to get the plasma membrane proteins by affinity chromatography and density gradient centrifugation with agents provided in Membrane Protein Extraction Kit. The plasma membrane and cytoplasm fractions were then dissolved in 0.5% Triton X-100 in PBS, and were bathed in 45°C with SDS sample buffer for 45 min for inactivation. In order to validate the effectiveness of membrane fraction and cytoplasm extraction, we examined the sodium-potassium adenosine triphosphatase (Na^+^/K ^+^ ATPase) which is considered as a specific marker of the plasma membrane in Western Blot by using its specific antibody. The result showed that under the same antibody concentration, the same exposure time, and the sample loading amount, a high intensity signal of sodium potassium ATPase was detected in the plasma membrane samples, while in the cytoplasm sample the signal is poor, which proves the effectiveness of our sample preparation (see Supplementary Figure S1 in supplementary data).

### Immunoblotting

Samples from above preparation were loaded and separated by SDS-PAGE, and latter electrophoretically transferred to Poly vinylidene fluoride (PVDF) membranes (Millipore). Loaded PVDF membranes were incubated with different primary antibodies in 5% skimmed milk—TBS-T solution (20 mM Tris, pH 7.6, 137 mM NaCl, 0.05% Tween 20) overnight at 4°C, followed by incubation with peroxidase-conjugated affinipure goat anti-rabbit (#111-035-003, 1:20,000; Jakson) or rabbit anti-goat (#305-035-003, 1:20,000; Jakson) secondary antibody in TBS-T buffer. Bands were visualized by using an ECL system (Pierce). The immunoreactivity of individual band was measured by Imagepro plus (IPP) were normalized to β-actin or GAPDH. The main-antibodies used in immunoblotting included: anti-KCC2 (#07-432, Millipore, 1:2,000), anti-m-calpain (#2539, CST, 1:1,000), anti-μ-calpain (#MAB3104, millipore, 1:1,000), anti-spectrin (#MAB1622, Millipore, 1:1,000), anti-β-actin (#94725S, CST, 1:2,000), anti-GAPDH (#ab8245, Abcam, 1:5,000).

### Immunoprecipitation

Hippocampal slices were incubated in 0 Mg^2+^ ACSF in the presence or absence of PD98059 (25 μM) or K252a (200 nM), then lysed and centrifuged (12,000 *g*, 5 min) in iced NP-40 lysis Buffer (beyotime, P0013F) containing protease inhibitor cocktail (#539134, Millipore), PMSF (1 mM), and phosphatase inhibitor (#4906845001, Sigma). Total amount of 10 μg μ-calpain (#MAB3104, Millipore) or m-calpain antibodies (#2539, CST) were added to Protein G Dynabeads (#10006D, Novex) for 30 min for cross-linking, then mixed with extraction from above and incubated overnight on a revolving rotor at 4°C to form antigen-antibody Dynabeads complex. The complex was washed multiple times with Washing Buffer (#10006D, Novex) to minimize nonspecific binding, and then eluted and separated on a Magnetic frame (#10006D, Novex). The supernatant was mixed with 5× loading buffer and heated for 10 min at 70°C for inactivation, and then processed for western blotting with anti-phosphor-serine antibody (#ab9332, Abcam).

### Power Spectrum Analysis

EEG data were exported to matlab (R2016a) as txt format and the recording channel was selected to generate a power spectrogram. The analysis script consisted of a fast Fourier transform using a Cosine-Bell data window with a window size of 1,024 data points. A window overlap of 87.5% was used to help to smooth the x-axis of the spectrogram. The power was expressed as μV^2^.

### Data Analysis

For normally distributed data, unpaired student’s *t*-tests was used for inter-group comparing, for not normally distributed data, we used Corresponding nonparametric analysis (i.e., Independent-Samples Mann-Whitney). Group data are expressed as mean ± SEM. Across different groups of data, statistically significant differences between means were determined using two-way ANOVA combined with Fisher’s LSD test. Comparison within a group was carried out using a paired or unpaired *t*-test. The significance level was set at *P* < 0.05.

## Results

### Calpain Inhibition Suppressed PTZ-Induced Acute Seizures

**Figure 1:**
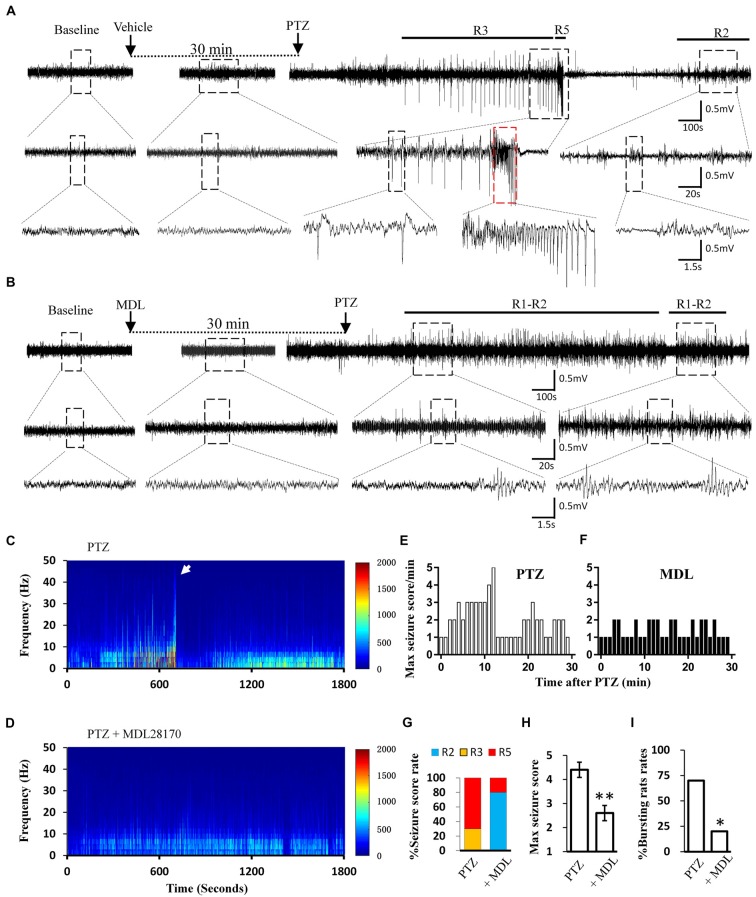
Electroencephalograph (EEG) and behavioral analysis showing improvements after MDL-28170 intervention on pentylenetetrazole (PTZ)-induced acute seizures. **(A,B)** typical EEG recordings from vehicle group **(A)** and MDL-28170 pre-treatment group **(B)** at selected time points. The lower traces are the enlarged traces from the dashed boxes of upper traces. The red dashed box marks an intermittent presence of high amplitude fast discharges (HAFDs) within the continuous rhythmic spike-wave activity. **(C,D)** EEG power from the recording of above traces was plotted against time, starting from the administration of PTZ and lasting for 30 min. The white arrow marks the HAFDs. **(E,F)** The static behavioral seizure scores over the 30 min period from two representative individual rats in the two groups illustrated in panel. **(G)** Proportion of rats with different seizure scores. R2, R3 and R5 represent the seizure score of Racine2, Racine3 and Racine5, respectively. **(H)** The average of the maximal behavior scores for rats from either PTZ or PTZ + MDL group. **(I)** Rate of animal with bursts and HAFDs within the two groups. *p < 0.05 compared with PTZ group; **p < 0.01 compared with PTZ group.

Calpain is involved in numerous neurological disorders, including seizure. Previous studies have demonstrated that calpain inhibitor MDL-28170 exerts suppressive effect on KA and pilocarpine induced seizure severity (Li et al., 2013; Lam et al., 2017). In current study, we further tested whether suppressing calpain activity with MDL-28170 would interfere the seizure activities in rats. We applied MDL-28170 to awake, freely moving rats 30 min prior to seizure induction with PTZ and simultaneously recorded seizure behavior and EEG patterns. Our result showed that MDL-28170 (50 mg/kg) significantly suppressed seizure behavior and epileptic activities. Seven out of 10 rats (70%) in the vehicle control group developed Racine-5 seizure behaviors after PTZ injection. Typical performances included twitching while standing and loss of balance. These behaviors were sometimes accompanied by systemic tonic convulsion. The EEG patterns of the rats showed highly synchronized cluster firing or burst firing (Figure 1A). Although the other rats did not develop Racine-5 behavior, they demonstrated unilateral limb clonus (Racine 3), and their EEG components were mainly characterized by spikes and high-amplitude slow waves at approximately 3 Hz. By contrast, only 20% (2 out of 10) of the rats in the MDL-28170 pretreated group showed Racine-5 seizure behavior and burst firing after PTZ injection. The seizure incidence in the MDL-28170 pretreated group was significantly lower than that in the control group (Figures 1G–I, *P* < 0.05). The rats that failed to demonstrate Racine-5 behavior only showed some Racine-2 behaviors, such as nodding and WDS, and their EEG patterns mainly presented low-frequency oscillations (Figure 1B). Time-frequency power analysis suggested that the firing patterns of these two groups differed after PTZ induction. In vehicle-PTZ animals, the intensity of high-frequency firing increased until HAFDs. This phase was followed by a defined silent phase, and regular spiking was later restored at approximately 10 Hz (Figure 1C). By contrast, most of (80%) the MDL-28170-pretreated animals exhibited low power but not HAFDs or a silent period during the recording (Figure 1D). Meanwhile, the real-time behavioral score per minute of the MDL pretreated rats during the whole recording course was significantly lower than that of the control PTZ group (Figures 1E,F). These results suggested that calpain is involved in seizure induction and calpain inhibitor could reduce the intensity and possibility of seizure induction in an animal model of epilepsy.

### Calpain Inhibitor MDL-28170 Prevented Convulsant-Stimulation-Evoked KCC2 Down-Regulation in the Hippocampus

**Figure 2:**
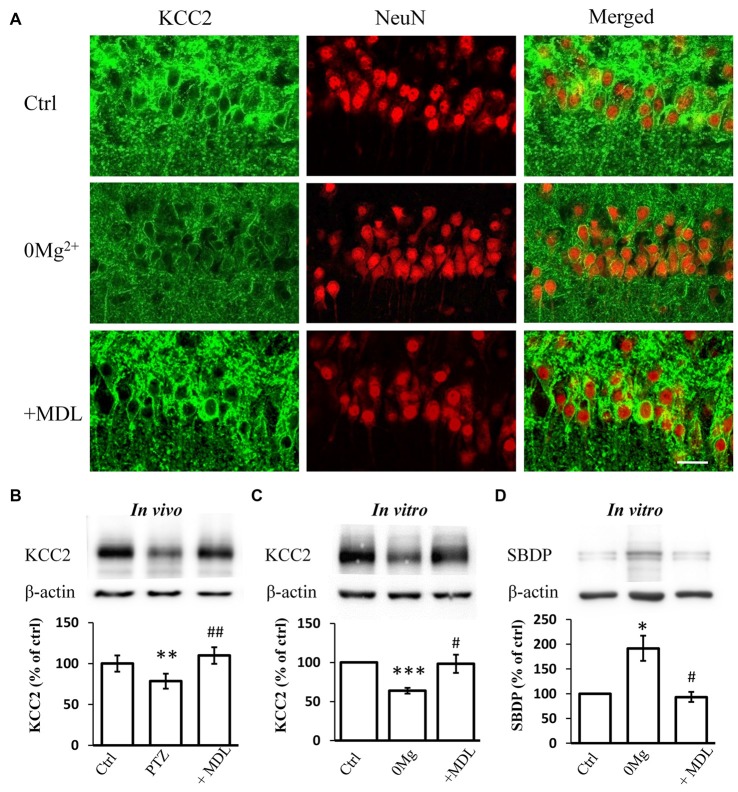
MDL-28170 reverses the down regulation of potassium chloride co-transporter 2 (KCC2) during seizure induction. **(A)** KCC2 immunostaining showing expression of KCC2 in membrane of hippocampal CA1 neurons after 0 Mg^2+^ (middle) or 0 Mg^2+^ + MDL-28170 (bottom) treatment, in comparison with vehicle control (left). Green and red signals represent the KCC2 and NeuN immunostaining, respectively. Scale bar: 20 μm. **(B)** The plasma membrane KCC2 (mKCC2) expression in hippocampus from PTZ alone or PTZ + MDL-21870 treated rats. The relative protein levels were normalized to the average of control group. **(C)** Spectrin breakdown product (mKCC2) expression from hippocampal slices. Slices were prepared from fresh brain and randomly divided into three different groups. KCC2 levels were normalized to control slices from the same preparation. **(D)** Spectrin breakdown product (SBDP)-145 level of 0 Mg^2+^ and 0 Mg^2+^ + MDL-28170 treated slices (total protein sample), normalized to control slices from the same preparation. *p < 0.05 compared with vehicle-PTZ group; **p < 0.01 compared with vehicle-PTZ group; ***p < 0.001 compared with vehicle-PTZ group; ^#^*p* < 0.05 compared with 0 Mg^2+^ group; ^##^*p* < 0.01 compared with 0 Mg^2+^ group.

Our previous work suggested that the decreased expression and function of KCC2 is necessary for epileptogenesis (Chen et al., 2017). The over-activation of calpain, a KCC2 decomposer, may contribute to the down-regulation of KCC2. Thus, we tested whether the rescuing effect of calpain inhibition on seizure susceptibility occurs through interference with KCC2 regulation. First, we performed immunostaining to examine KCC2 expression levels in hippocampal slices incubated with 0 Mg^2+^ ACSF. We found that blocking calpain activity by MDL-28170 (50 μM) reversed 0 Mg^2+^-induced impairment of fluorescence intensity (Figure 2A). We further performed immunoblotting to quantify the expression level of mKCC2 in hippocampal slices from the *in vivo* PTZ model and *in vitro* 0 Mg^2+^ model. Our results showed that mKCC2 was significantly down-regulated in the *in vivo* PTZ and *in vitro* 0 Mg^2+^ models but was significantly reversed in the MDL-28170 pretreatment group (*in vivo*: PTZ: 78.5 ± 9.2%, *n* = 8; PTZ + MDL-28170: 109.8 ± 10.1%, *n* = 8. PTZ vs. Vehicle: *P* < 0.01; PTZ vs. PTZ + MDL-28170: *P* < 0.01; Figure 2B; *in vitro*: 0 Mg^2+^: 73.4 ± 4.1%, *n* = 6; 0 Mg^2+^ + MDL-28170: 94.1 ± 7.8%, *n* = 6. 0 Mg^2+^ vs. Vehicle: *P* < 0.001; 0 Mg^2+^ vs. 0 Mg^2+^ + MDL-28170: *P* < 0.05; Figure 2C). These results indicated there is abnormal over-activation of calpain during seizure onset.

We further studied whether convulsant stimulation induced calpain over-activation by quantifying the levels of the 145 KD spectrin breakdown product (SBDP-145), which can be used as an indicator of calpain activity (Kupina et al., 2001; Zadran et al., 2010). Indeed, we detected significantly elevated SBDP-145 levels in hippocampal slices from the 0 Mg^2+^ model. As expected, this increase was reversed by co-treatment with MDL-28170 (50 μM; 0 Mg^2+^: 187.2 ± 15.4%, *n* = 3; 0 Mg^2+^ + MDL-28170: 99.5 ± 8.4%, *n* = 3. 0 Mg^2+^ vs. Vehicle: *P* < 0.05; 0 Mg^2+^ vs. 0 Mg^2+^ + MDL-28170: *P* < 0.05; Figure 2D). These results indicated that the application of calpain inhibitors in both *in vitro* and *in vivo* epilepsy models can reverse the convulsant induced KCC2 down-regulation.

### M-Calpain but Not μ-Calpain Involved in KCC2 Regulation During Seizure Induction

**Figure 3:**
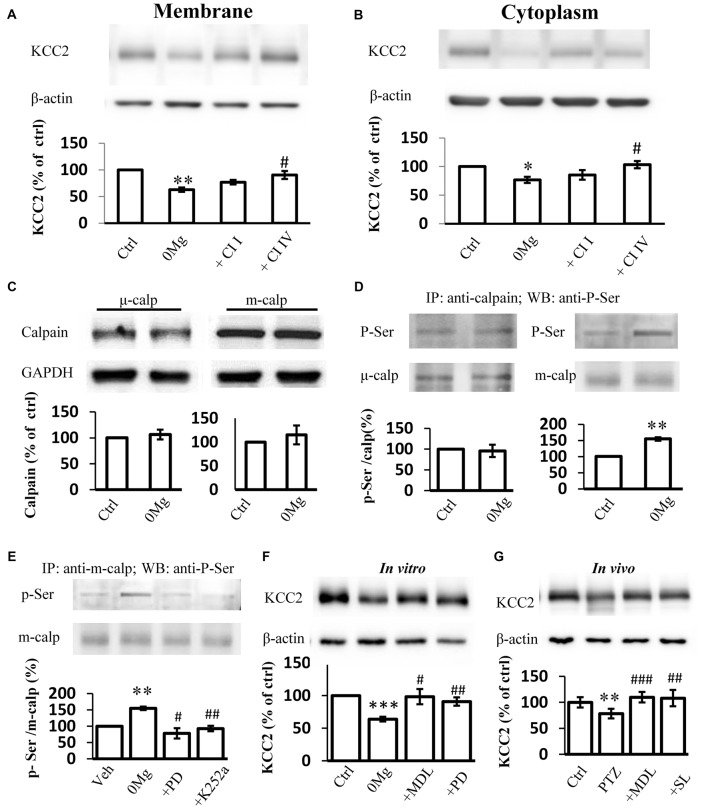
Phosphor-serine activation of m-calpain dominants KCC2 regulation in 0 Mg^2+^ seizure model. **(A,B)** Immunoblotting detection of both plasma membrane **(A)** and cytoplasm **(B)** KCC2 expression level change in hippocampal slice 0 Mg^2+^ model treated with either μ-calpain or m-calpain selective inhibitor CI I and CI IV, respectively. **(C)** Expression of either μ- or m-calpain from seizure slices induced by 0 Mg^2+^. **(D)** Serine phosphorylation level by IP method. Naïve or 0 Mg^2+^ treated slices were used as inputs to form immune complex with μ- or m-calpain antibodies and detected by phosphor-serine antibody. Phosphor-serine levels were normalized to corresponded calpain levels. **(E)** Phosphor-serine level of m-calpain from 0 Mg^2+^, 0 Mg^2+^ + PD98059 or K252a treated slices. **(F)** Immunoblotting showed KCC2 level in 0 Mg^2+^ + MDL-28170 or PD98059 treated slices, compared with none treated or 0 Mg^2+^ treated alone slices. **(G)** KCC2 level of *in vivo* rats pre-treated with either MDL-28170 or SL-327 before PTZ kindling, in compared with vehicle PTZ treated rats. CI I, Calpain Inhibitor I; CI IV, Calpain Inhibitor IV; Calp, calpain; p-Ser, Phosphor-serine. *p < 0.05 compared with Ctrl group; **p < 0.01 compared with Ctrl group; ***p < 0.001 compared with Ctrl group; ^#^*p* < 0.05 compared with 0 Mg^2+^ group; ^##^*p* < 0.01 compared with either 0 Mg^2+^ or with PTZ group; ^###^*p* < 0.001 compared with PTZ group.

Two main calpain isoforms exist: μ-calpain and m-calpain. These isoforms primarily differ in their required Ca^2+^ concentration for activation (Glass et al., 2002). The contributions of these isoforms to KCC2 regulation remain unknown. Thus, we applied either 100 μM Calpain Inhibitor I, a μ-calpain-specific inhibitor (Griscavage et al., 1996; Gellerman et al., 1997) or 200 μM Calpain Inhibitor IV, a m-calpain-specific inhibitor (Rosenberger et al., 2005), in 0 Mg^2+^ model to identify which isoform that contributes to the change of KCC2 expression during convulsant stimulation. Our results showed that the expression of KCC2 on plasma membrane in hippocampal slices from the 0 Mg^2+^ model recovered to the control level after treatment with Calpain Inhibitor IV (200 μM) but not after treatment with Calpain Inhibitor I (100 μM; 0 Mg^2+^: 62.8 ± 4.0%, *n* = 4; 0 Mg^2+^ + Cl I: 77.0 ± 4.4%, *n* = 4; 0 Mg^2+^ + Cl IV: 90.4 ± 7.3%, *n* = 4. 0 Mg^2+^ vs. Vehicle: *P* < 0.01; 0 Mg^2+^ vs. 0 Mg^2+^ + Cl I: *P* > 0.05; 0 Mg^2+^ vs. 0 Mg^2 +^ + Cl IV: *P* < 0.05; Figure 3A). Changes in cytoplasmic KCC2 (cKCC2) expression are similar to those in mKCC2 expression (0 Mg^2+^: 76.7 ± 5.4%, *n* = 4; 0 Mg^2+^ + Cal I: 85.3 ± 9.1%, *n* = 4; 0 Mg^2+^ + Cal IV: 103.3 ± 6.3%, *n* = 4; 0 Mg^2+^ vs. 0 Mg^2+^ + Cal I: *P* > 0.05; 0 Mg^2+^ vs. 0 Mg^2+^ + Cal IV: *P* < 0.05; Figure 3B). These results collectively indicated that it was m-calpain, rather than μ-calpain, contributed to KCC2 regulation during seizure induction.

### Phosphor–Serine M-Calpain Regulated Cellular KCC2 Level During Seizure Induction

The enhancement in calpain activity after seizure induction with 0 Mg^2+^ may be attributed to increases in calpain expression levels. Therefore, we examined the expression levels of both m-calpain and μ-calpain. Surprisingly, neither m- nor μ-calpain altered during seizure induction with 0 Mg^2+^ (m-calpain: 106.5 ± 9.5%, *n* = 3, μ-calpain: 103.4 ± 8.8%, *n* = 3; 0 Mg^2+^ vs. Vehicle: *p* > 0.5; Figure 3C). This result suggested that enhanced calpain activation after seizure induction with 0 Mg^2+^ was not resulted from changing of either m- or μ-calpain expression. Given that calpain expression levels remained unchanged, we focused on alteration of calpain function and activity. As previously reported, calpain can be activated through the phosphorylation of its Ser50 site (Glading et al., 2004; Zadran et al., 2010). We therefore examined the serine phosphorylation levels of both m-calpain and μ-calpain after epileptic induction with 0 Mg^2+^. After brain homogenate extracts were incubated with the main m-calpain or μ-calpain antibody to form immune complexes then subjected to immunoprecipitation and immunoblotting with an anti-serine phosphorylation antibody, we found that the phosphorylation rate of μ-calpain was not significantly changed, whereas that of m-calpain was significantly increased (μ-calpain: 95.8 ± 10.5%, *n* = 3, *P* > 0.05; m-calpain: 154.6 ± 5.2%, *n* = 3, *P* < 0.01; Figure 3D).

It is reported that in cultured primary neurons and HEK-TrkB cells, phosphorylation of calpain Ser50 site is regulated by the up-stream MAPK/ERK pathway (Glading et al., 2004; Zadran et al., 2010). However, whether MAPK/ERK pathway phosphorylation of Ser50-calpain participates in seizure onset needs to be verified. We treated 0 Mg^2+^- hippocampal slices with the ERK1-specific inhibitor PD98059. We then examined the phosphor-serine levels of the treated slices through IP. Our result showed that PD98059 (25 μM) eliminated the 0 Mg^2+^-induced up-regulation of the phosphor–serine level of m-calpain (0 Mg^2+^: 154.6 ± 5.2%, *n* = 3; 0 Mg^2+^ + PD98059: 78.1 ± 15.8%, *n* = 3; 0 Mg^2+^ vs. Vehicle: *P* < 0.01; 0 Mg^2+^ vs. 0 Mg^2+^ + PD98059: *P* < 0.05 (Figure 3E). ERK1 is regulated by the BDNF/TrkB signaling pathway (Mocchetti and Bachis, 2004; Numakawa et al., 2010), and the TrkB signaling pathway is activated during seizure induction has been demonstrated in varies epilepsy animal models including PTZ model (Wang et al., 2009; Liu et al., 2013; Hao et al., 2016; Enomoto et al., 2017). To confirm the participation of the BDNF/TrkB pathway regulating ERK-calpain activity in epileptogenesis, we applied K252a (200 nM), a TrkB antagonist, and found that the phosphorylation level of m-calpain in the K252a-treated group returned to normal level which is significantly reduced in comparison with that in the 0 Mg^2+^ group (0 Mg^2+^: 154.6 ± 15.2%, *n* = 3; 0 Mg^2+^ + K252a: 92.8 ± 28.2%, *n* = 3. Vehicle vs. 0 Mg^2+^ + K252a: *P* > 0.05; 0 Mg^2+^ vs. 0 Mg^2+^ + K252a: *P* < 0.01; Figure 3E). Thus, we demonstrated that the TrkB-MAPK/ERK pathway regulates the phosphor–serine activation of m-calpain during seizure induction. Nevertheless, the contribution of this mechanism to KCC2 regulation is still not clear. We then tested the KCC2 levels in PD98059 treated 0 Mg^2+^ model of hippocampal slices, and also directly compared with MDL-28170 treatment. The results showed that, similar to MDL-28170, PD98059 alone also restored KCC2 expression to levels close to those in the vehicle control group, but significantly higher than those in 0 Mg^2+^ group. Furthermore, the KCC2 levels between PD98059- and MDL-28170-treated groups were not different (0 Mg^2+^: 63.9 ± 3.6%, *n* = 6; 0 Mg^2+^ + MDL-28170: 98.3 ± 11.7%, *n* = 6; 0 Mg^2+^ + PD98059: 90.7 ± 6.4%. 0 Mg^2+^ vs. Vehicle: *P* < 0.001; 0 Mg^2+^ + MDL-28170 vs. 0 Mg^2+^: *P* < 0.05; 0 Mg^2+^ + PD98059 vs. 0 Mg^2+^: *P* < 0.01; 0 Mg^2+^ + PD98059 vs. 0 Mg^2+^ + MDL-28170: *P* > 0.05; Figure 3F). Furthermore, we also validated this effect in rats with PTZ-induced seizures. Given that PD98059 cannot pass through the blood–brain barrier, we used SL-327, a brain penetrable ERK1 blocker for *in vivo* validation. The results demonstrated that, after blocking m-calpain phosphorylation with SL-327 (50 mg/kg, i.p.), KCC2 expression level was rescued to vehicle control level, but significantly higher than that in the PTZ seizure group. Again, the KCC2 expression levels of SL-327-treated animals were not significantly different from those of MDL-28170-treated animals (PTZ + Vehicle: 78.5 + 9.2%, *n* = 8; PTZ + MDL-28170: 109.8 + 10.1%, *n* = 8; PTZ + SL-327: 108.1 + 15.6%, *n* = 8. PTZ + Vehicle vs. saline + Vehicle: *P* < 0.01; PTZ + Vehicle vs. PTZ + MDL-28170: *P* < 0.001; PTZ + Vehicle vs. PTZ + SL-327: *P* < 0.01; PTZ + MDL-28170 vs. PTZ + SL-327: *P* > 0.05; Figure 3G).

These results suggested that m-calpain, but not μ-calpain, is over-activated as a result of its serine site phosphorylation by the activation of TrkB–MAPK/ERK signaling pathway, which in turn plays a major role in the regulation of KCC2 expression during seizure induction.

### MAPK/ERK Signaling Pathway Induced M-Calpain Phosphorylation and KCC2 Down-Regulation Is Independent of [Ca^2+^]_i_

**Figure 4:**
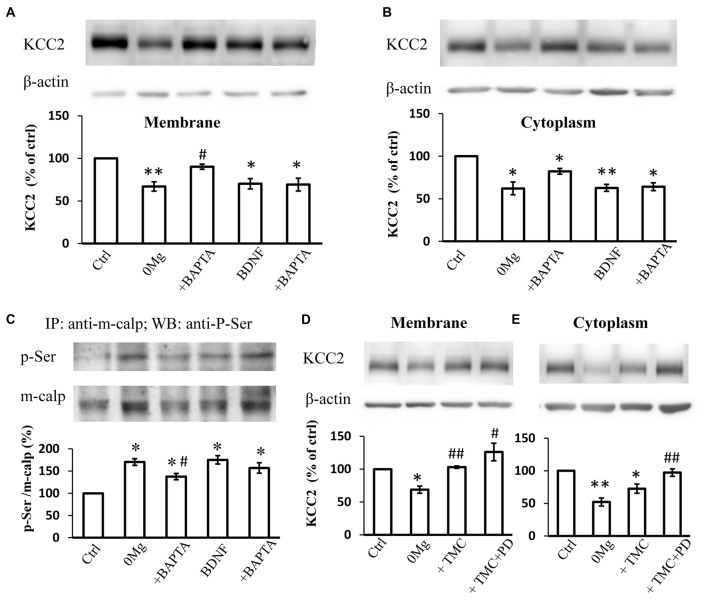
The regulation of KCC2 by m-calpain is independent on [Ca^2+^]_i_ but is related to KCC2 endocytosis. **(A)** BAPTA is able to reverse 0 Mg^2+^ induced mKCC2 down-regulation, but failed to reverse BDNF induced mKCC2 down-regulation. **(B)** BAPTA partially reverses the 0 Mg^2+^ induced, but not BDNF induced Changes in cytoplasmic KCC2 (cKCC2) down-regulation. **(C)** Phosphor-serine level of m-calpain from 0 Mg^2+^, 0 Mg^2+^ + BAPTA, BDNF and BDNF + BAPTA treated slices. BAPTA intervention partially reverses the 0 Mg^2+^ induced, but not BDNF induced the increase of phosphor-serine level of m-calpain. **(D–E)** Tautomycetin (TMC) can reverse KCC2 down-regulation in plasm membrane (left), but the expression of KCC2 in cytoplasm remained significantly lower than DMSO group (right). In TMC + PD98059 co-treated group the expression of KCC2 is rescued to control level. *p < 0.05, **p < 0.01 compared with Ctrl group; ^#^*p* < 0.05, ^##^*p* < 0.01 compared with either 0 Mg^2+^ group.

Since both m-calpain and μ-calpain activity is sensitive to [Ca^2+^]_i_, we therefore studied whether seizure induced phosphor-serine of m-calpain involves with [Ca^2+^]_i_ change. BAPTA-AM, an endogenous Ca^2+^ chelating agent, was used in our study to exclude the effect of the changes in [Ca^2+^]_i_ during seizure induction. The results showed that, in BAPTA (10 μM) treated 0 Mg^2+^ hippocampal slice model, the mKCC2 level reversed significantly when compared to 0 Mg^2+^ group, and no difference compared with control group (0 Mg^2+^: 67.1 ± 5.5%, *n* = 4; 0 Mg^2+^ + BAPTA: 90.3 ± 3.1%. 0 Mg^2+^ vs. Vehicle: *P* < 0.05; 0 Mg^2+^ + BAPTA vs. Vehicle: *P* > 0.05; 0 Mg^2+^ vs. 0 Mg^2+^ + BAPTA: *P* = 0.05)(Figure 4A). Meanwhile BAPTA failed to fully reverse the 0 Mg^2+^ induced cKCC2 down-regulation, but reduced the 0 Mg^2+^ evoked cKCC2 reduction by almost 50% (0 Mg^2+^: 62.2 ± 7.5%, *n* = 4; 0 Mg^2+^ + BAPTA: 82.2 ± 3.3%. 0 Mg^2+^ vs. Vehicle: *P* < 0.05; 0 Mg^2+^ + BAPTA vs. Vehicle: *P* < 0.05; 0 Mg^2+^ vs. 0 Mg^2+^ + BAPTA: *P* = 0.07; Figure 4B).

Since BDNF can directly activate the MAPK/ERK signaling pathway (Numakawa et al., 2010), which leads to the activation of phosphorylation of m-calpain, we also tested whether [Ca^2+^]_i_ involved in BDNF-TrkB-MAPK/ERK signaling pathway in regulation of m-calpain phosphorylation. We applied BDNF to activate the down-stream MAPK/ERK pathway in normal ACSF with or without BAPTA-AM treatment, and measured both plasma membrane and cytoplasm KCC2 expression change. Our results showed that BAPTA-AM was not able to reverse the BDNF-induced down-regulation of either mKCC2 or cKCC2 (mKCC2: BDNF: 70.2 ± 6.0%, *n* = 4; BDNF + BAPTA: 69.3 ± 7.5%. BDNF vs. Vehicle: *P* < 0.05; BDNF + BAPTA vs. Vehicle: *P* < 0.05; BDNF vs. BDNF + BAPTA: *P* > 0.05; Figure 4A; cKCC2: BDNF: 62.8 ± 4.1%, *n* = 4; BDNF + BAPTA: 64.2 ± 4.6%. BDNF vs. Vehicle: *P* < 0.01; BDNF + BAPTA vs. Vehicle: *P* < 0.05; BDNF vs. BDNF + BAPTA: *P* > 0.05; Figure 4B).

Next, we examined the changes in m-calpain phosphorylation levels after BAPTA-AM treatment in both 0 Mg^2+^ and BDNF induced model. The results demonstrated that the increased phosphorylation level of m-calpain induced by 0 Mg^2+^ significantly decreased when co-treated with BAPTA-AM (0 Mg^2+^: 170.3 ± 7.6%, *n* = 3; 0 Mg^2+^ + BAPTA: 137.5 ± 7.1%, *n* = 3. 0 Mg^2+^ + BAPTA vs. 0 Mg^2+^: *P* < 0.05; Figure 4C), but still significantly higher than that of the control group (*P* < 0.05). In contrast, although BDNF also induced the increase of m-calpain phosphorylation level, BAPTA-AM treatment failed the affect the BDNF induced m-calpain phosphorylation (BDNF: 175.2 ± 9.4%, *n* = 3; BDNF + BAPTA: 157.0 ± 11.8%, *n* = 3. BDNF vs. Vehicle: *P* < 0.05; BDNF + BAPTA vs. Vehicle: *P* < 0.05; BDNF vs. BDNF + BAPTA: *P* > 0.05; Figure 4C). Putting together, these results demonstrated that, in 0 Mg^2+^ model, both the mKCC2 level and the m-calpain phosphor-serine level were significantly affected by the cleavage of intracellular Ca^2+^, while neither KCC2 level nor phosphorylation of m-calpain induced by BDNF were affected by BAPTA. The data suggested that BDNF-TrkB-MAPK/ERK signaling pathway in regulation of m-calpain phosphorylation and in turn regulation of cellular KCC2 level is likely independent of [Ca^2+^]_i_. Since m-calpain phosphorylation and cellular KCC2 regulation are likely more complicated with multiple mechanism, our data also suggested that, at least with some parts, this process is dependent on [Ca^2+^]_i_ level during seizure induction in 0 Mg^2+^ epilepsy model.

### KCC2 Degradation Mediated by Phosphor–Serine-Activated M-Calpain Is Related to KCC2 Endocytosis

mKCC2 regulation is a complex and dynamic process that comprises endocytosis and membrane transport (Lee et al., 2007; Chamma et al., 2013; Medina et al., 2014). The stability of mKCC2 can be regulated by the dephosphorylation of its 940 serine site, and the dephosphorylation of membrane KCC2 is regulated by protein phosphatase 1 (PP1; Lee et al., 2007, 2011; Silayeva et al., 2015). To understand the relationship between KCC2 endocytosis and m-calpain-mediated KCC2 degradation, we blocked the dephosphorylation of the ser940 site of KCC2 and prevented the internalization of mKCC2 in 0 Mg^2+^-treated hippocampal slices by applying the PP1-specific inhibitor tautomycetin (TMC). Compared with treatment with DMSO or 0 Mg^2+^ alone, application of TMC (20 nM) alone or combined with PD98059 all significantly reversed the down-regulation of mKCC2 (0 Mg^2+^: 68.7 ± 5.5%, *n* = 4; 0 Mg^2+^ + TMC: 103.1 ± 1.8%, *n* = 4; 0 Mg^2+^ + TMC + PD98059: 126.1 ± 13.3%, *n* = 4. 0 Mg^2+^ vs. Vehicle: *P* < 0.05; 0 Mg^2+^ + TMC vs. 0 Mg^2+^: *P* < 0.01; 0 Mg^2+^ + TMC + PD98059 vs. 0 Mg^2+^: *P* < 0.05; Figure 4D). However, TMC treatment alone to block KCC2 internalization failed to reverse the cKCC2 levels after 0 Mg^2+^ stimulation, however, co-treatment with PD98059 significantly recovered cKCC2 to normal levels (0 Mg^2+^: 52.2 ± 6.2%, *n* = 4; 0 Mg^2+^ + TMC: 72.6 ± 7.1%, *n* = 4; 0 Mg^2+^ + TMC + PD98059: 97.2 ± 5.8%, *n* = 4; 0 Mg^2+^ vs. Vehicle: *P* < 0.01; 0 Mg^2+^ + TMC vs. 0 Mg^2+^: *P* > 0.05; 0 Mg^2+^ + TMC vs. Vehicle: *P* < 0.05; 0 Mg^2+^ +TMC + PD98059 vs. 0 Mg^2+^: *P* < 0.01; Figure 4E). These results revealed that the seizure-induced enhancement of KCC2 endocytosis will further stimulate the m-calpain activation, therefore, enhance the m-calpain-mediated KCC2 degradation.

### Blocking the MAPK/ERK Signaling Pathway Alleviated Acute Seizures *in vivo*

**Figure 5:**
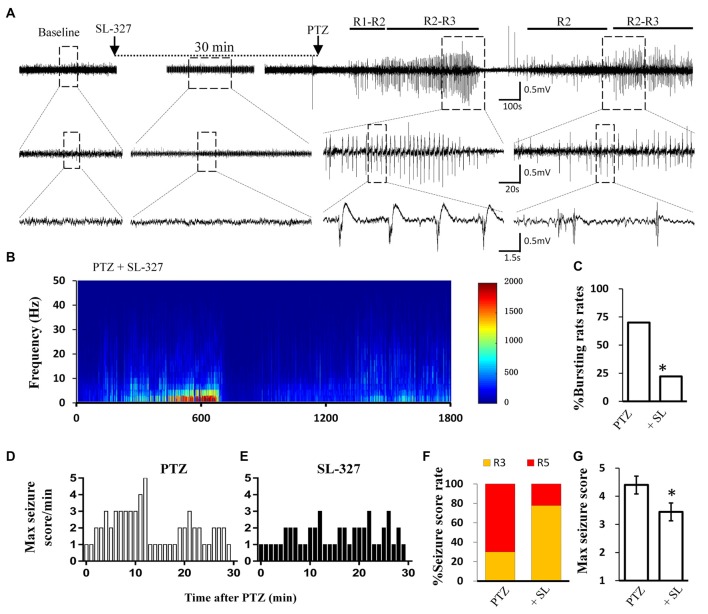
SL-327 intervention improved the incoming seizure induced by PTZ *in freely moving rats*. **(A)** Typical EEG recording traces from SL-327 pre-treated rats. The lower traces are an enlarged drawing from the dashed boxes of upper traces. **(B)** EEG power from the recording of above trace was plotted against time, from PTZ administration to the following 30 min. The intensity of high frequency firing has been increasing in the first 10 min, but never reached to HAFDs during 30 min analysis in comparison with PTZ control rat described in Figure 1A. **(C)** Rate of animal with bursts and HAFDs from the two treating groups. **(D,E)** The static behavioral seizure scores over the 30 min period from the PTZ alone and PTZ + SL-327 groups illustrated in panel. **(F)** Proportion of rats with different seizure score. R3 and R5 represent the seizure score of Racine3 and Racine5, respectively. **(G)** The average of the maximal behavior scores for rats from either PTZ or SL-327 group. *p < 0.05 compared with PTZ group.

Similar to direct calpain inhibition by MDL-28170, MAPK/ERK blockade also reversed the reduction of KCC2 during seizure stimulation. Thus, we next tested whether blockade of the MAPK/ERK pathway would also have similar effects as MDL-28170 on seizure induction *in vivo*. Given that PD98059 cannot penetrate the blood–brain barrier, we applied the brain penetrable ERK1 inhibitor SL-327 (Carr et al., 2009) to abolish the serine-site phosphorylation of m-calpain and recorded PTZ induced seizure behavior and the EEG patterns in rats. Our results showed that the proportion of rats with Racine-5 seizure behaviors and HDFAs in the SL-327 pretreatment group was significantly lower than that in the Vehicle-PTZ group, as described in the previous section (Figures 1F,G, 5F,G; *P* < 0.05). Two out of 10 rats in the SL-327 pretreatment group presented Racine-5 seizure behaviors and EEG patterns with bursts and HDFAs. Eight of the remaining rats developed only Racine-3 behaviors (Figure 5E), and their EEG patterns were mainly characterized by slow waves mixed with 0–10 Hz spikes (Figure 5A). The fact that SL-327 mimicked the anti-seizure effect of complete calpain blockade suggested that the activation of m-calpain phosphorylation by the MAPK/ERK signaling pathway is the dominant mechanism of calpain involvement in epilepsy in the mechanism of calpain involved in epilepsy.

## Discussion

Our research indicates that, during the seizure onset, the over-activation of m-calpain, but not μ-calpain, enhances KCC2 degradation, and in turn, results in the functional down-regulation of KCC2 on the plasma membrane. Enhanced m-calpain activation during seizure induction is due to the over phosphorylation by the MAPK/ERK signaling pathway activity. Inhibition of either calpain or MAPK/ERK activities significantly suppressed seizure induction *in vivo*. Since our previous study (Chen et al., 2017) and studies of other researchers (Huang et al., 2012; Baek et al., 2016) have already shown that KCC2 is a key protein in the onset of seizure, and its functional down-regulation plays a decisive role in the formation and development of epileptiform neuronal bursting discharges and subsequent epilepsy, our current study may provide a possible mechanism that enhanced m-calpain activation during convulsant stimulation facilitates seizure induction by increasing KCC2 down-regulation.

Our *in vivo* epileptic model study found that the nonselective calpain blocker MDL-28170 significantly inhibited the onset of seizures in PTZ rat model, which is well in agree with previous reported that inhibition of calpain by MDL-28170 could reduce seizure probability and severity in both kainate acid and pilocarpine induced seizure animal models (Sierra-Paredes et al., 1999; Araujo et al., 2008; Nam et al., 2017). PTZ induced seizure animal model is one of the widely used animal models for epilepsy study and drug screening. Our own previous studies have found that PTZ induced seizure behaviors and epileptiform bursting neuronal activities are relatively simple in its form and easy to be characterized (Qian et al., 2011; Liu et al., 2013). Thus, classic PTZ rat model has been used in this study. However, although PTZ has been widely accepted as a GABA_A_ receptor antagonist, the mechanism of PTZ peripheral injection to induce epilepsy is very complicated, and to our knowledge, it is not well defined so far. PTZ has been reported acts not only as a competitive inhibitor for GABA receptors, but also has been suggested to induce epileptic seizures through other mechanisms. A 1987 study found that PTZ acts at calcium channels, and it causes calcium channels to lose selectivity and conduct sodium ions as well (Papp et al., 1987). One other study pointed out the involvement of cAMP and its downstream effects upon PTZ activity (Hosseini-Zare et al., 2011). In addition, our own unpublished data showed that PTZ, not like other selective GABA_A_ receptor antagonists, failed to evoked epileptiform burst discharges *in vitro* in hippocampal slices, and was also not able to induce seizure behavior by intracerebroventricular (i.c.v.) injection (data not presented). Thus, we chose PTZ rat model to study the mechanism underlying KCC2 down regulation and GABA receptor dysfunction in current study. Indeed, we also found in current study that, similar as in other seizure animal models, PTZ induced seizure behaviors are closed correlated to the down regulation of the mKCC2 (Huang et al., 2012; Chen et al., 2017). In this study, we found that calpain inhibitor MDL-28170 could also prevent either *in vivo* PTZ induced or *in vitro* 0 Mg^2+^ induced KCC2 down regulation. Thus, the antiepileptic effect of MDL-28170 may be attributed to the regulation of KCC2, since KCC2 down regulation during convulsant stimulation has been closely associated to the seizure onset (Huang et al., 2012; Baek et al., 2016; Chen et al., 2017). Furthermore, this antiepileptic action of calpain inhibition is likely resulted from blockade of m-calpain rather than from modulation of μ-calpain, since selective m-calpain selective inhibitor Calpain Inhibitor IV (Griscavage et al., 1996; Gellerman et al., 1997; Rosenberger et al., 2005), but not μ-calpain selective inhibitor Calpain Inhibitor I (Griscavage et al., 1996; Gellerman et al., 1997), prevented 0 Mg^2+^ induced KCC2 down regulation (see Figures 3A,B,D).

Calpain is a Ca^2+^-dependent cysteine protease that is ubiquitously expressed in mammals (Zimmerman and Schlaepfer, 1984; Ono et al., 2016), and two major subtypes of calpain have different sensitivity to the Ca^2+^ concentration with μ-calpain at about 3–50 μM Ca^2+^ and, distinguishably, m-calpain at around 400–800 μM, respectively (Liu et al., 2008; Sorimachi et al., 2011a; Zanardelli et al., 2013). It is reported that, at normal condition, intracellular Ca^2+^ concentration is at around 50–300 nM and extracellular Ca^2+^ concentration is approximately at around 2 mM (Maravall et al., 2000), and pathological stimulation, such as convulsant, may trigger the Ca^2+^ influx and the release of Ca^2+^ from the endoplasmic reticulum into cytoplasm, to a level of hundreds of nM (Ono et al., 2016). Thus, convulsant stimulation induced intracellular Ca^2+^ concentration ([Ca^2+^]_i_) increase to a level which may be sufficient to activate μ-calpain, but may not enough to activate m-calpain. Since our pharmacological results, by using selective inhibitors for either m- or μ-calpain, indicated, m-calpain, but not μ-calpain, is likely to involve in convulsant stimulation induced KCC2 down regulation, it suggests that increases of [Ca^2+^]_i_ might only contribute minimal effect on calpain mediated KCC2 down regulation in *in vitro* 0 Mg^2+^ model or *in vivo* PTZ model used in our current study. Meanwhile, seizure stimuli such as 0 Mg^2+^ or PTZ would also causes the release of BDNF (Wang et al., 2009; Liu et al., 2013), which activates the MAPK/ERK pathway, in turn, could lead to the phosphorylation of the downstream m-calpain serine site (Chen et al., 2010; Liu et al., 2016). Our results indeed showed that K252a, a BDNF receptor TrkB receptor blocker, and PD98059, a MAPK/ERK inhibitor, both fully reversed enhanced m-calpain phosphorylation during 0 Mg^2+^ stimulation. Thus, our results suggest that MAPK/ERK-mediated m-calpain phosphorylation activation may be responsible for excessive calpain activation to modulate KCC2 expression under convulsant stimulation conditions and causes seizure induction.

The results from our current study indicate that, during seizure induction, activation of m-calpain, but not μ-calpain, promotes KCC2 down regulation. Since KCC2 down regulation is essential for seizure onset (Huang et al., 2012; Baek et al., 2016; Chen et al., 2017), the result that μ-calpain has little effect on KCC2 regulation might suggest that μ-calpain has relatively little contribution to seizure development. In fact, recent studies on different pathological models have revealed that the two main calpain subtypes have different functions in response to pathological stimuli. For example, μ-calpain and m-calpain play different and even opposing roles in TBI, stroke, and neurodegenerative diseases (Puskarjov et al., 2015; Etehadi Moghadam et al., 2018; Wang et al., 2018b). Generally speaking, m-calpain activation promotes neuronal apoptosis and aggravates neurotoxicity, whereas μ-calpain activation provides neuroprotective effects and relieves pathological stimulation (Baudry and Bi, 2016; Wang et al., 2018a). In present study, we have proved that m-calpain is the dominant participant in seizure onset, by regulating of KCC2 level. Besides m-calpain and μ-calpain, whom also called traditional calpain, the other calpain subtypes, which are called non-traditional calpain (Sorimachi et al., 2011b), have been paid little attention of their role in pathological condition. Whether these non-traditional calpain subtypes also contribute to the seizure onset needs to be studied in future.

KCC2 has a high turnover rate at about 50% renewal rates during 10 min period (Lee et al., 2007; Zhao et al., 2008). Under physiological conditions, normal calpain activation sustains the dynamic balance of KCC2 transportation and degradation. By contrast, under pathological conditions, such as seizure onset, calpain is excessively activated to scavenge cKCC2. The over-activation of calpain degrades KCC2 and in turn diminishes the intracellular KCC2 pool, an effect that eventually decreases KCC2 expression in the plasma membrane (Puskarjov et al., 2012, 2015; Zhou et al., 2012; Chamma et al., 2013). The stability of KCC2 on the plasma membrane is affected by the phosphorylation of its ser940 site, which can be dephosphorylated by PP1 (Lee et al., 2007, 2011; Silayeva et al., 2015). In our current study, after the internalization of mKCC2 was prevented by blocking the dephosphorylation of the ser940 site of membrane KCC2 with TMC, our results, however, showed that although the down-regulation of mKCC2 was rescued, the cKCC2 level was still at low level (see Figure 4). It suggested that calpain might still remain in over activation status in functioning of cleavage of KCC2. Indeed, our result further demonstrated that KCC2 down-regulation was completely restored after MAPK/ERK blockade by PD98059. These results indicated that internalized KCC2 will be further degraded by calpain activation during seizure induction. The phosphor-activation of m-calpain may function as a quick response to accumulated cKCC2.

As discussed above, it is likely during convulsant stimulation, that the enhanced m-calpain activation is due to MAPK/ERK phosphorylation of serine-site of m-calpain. We further tested, *in vivo*, whether inhibition of MAPK/ERK pathway could also suppress seizure induction, as well as interference of KCC2 down regulation. Indeed, SL-327, a brain penetrable ERK inhibitor (Carr et al., 2009), significantly blocked PTZ evoked KCC2 reduction and simultaneously prevented PTZ induced seizure behaviors in rats (see Figures 3;  5). Thus, our results that inhibition of m-calpain, as well as its up-stream regulator MAPK/ERK signaling pathway could block both KCC2 down regulation and seizure induction by convulsant stimulation, indicated MAPK/ERK—m-calpain pathway is one of the important regulatory pathways, by regulating the KCC2 expression level, hence influence the GABA inhibitory efficiency, to modulate seizure onset.

Since calpain is a Ca^2+^-dependent cysteine protease, whether intracellular Ca^2+^ concentration change during convulsant stimulation would affect m-calpain over activation is an important issue. Our BAPTA experiment results demonstrated that, in 0 Mg^2+^ model, both the KCC2 level and the m-calpain phosphor-serine level were significantly affected by the cleavage of intracellular Ca^2+^, while neither KCC2 level nor phosphorylation of m-calpain induced by BDNF were affected by BAPTA. These data suggested that, during seizure induction, over release of BDNF triggered TrkB-MAPK/ERK signaling pathway in regulation of m-calpain phosphorylation and in turn regulation of cellular KCC2 level is likely independent of [Ca^2+^]_i_. However, since m-calpain phosphorylation and cellular KCC2 regulation are likely more complicated regulated with multiple mechanisms, our data from 0 Mg^2+^ model also suggested that, at least with some parts, this process is dependent on [Ca^2+^]_i_ level during seizure induction in 0 Mg^2+^ or many other epilepsy models.

Although our current study showed that both non-selective calpain inhibitor MDL-28170 and MAPK/ERK inhibitor SL-327 all significantly inhibited acute epileptic seizures, it is worth paying attention to whether these targets are suitable for epileptic seizure treatment. Since m-calpain and μ-calpain have different or even opposite function (Puskarjov et al., 2015; Etehadi Moghadam et al., 2018; Wang et al., 2018b), the non-selective inhibition of calpain by using non-selective drugs such as MDL-28170 may cause serious side effects. The study in the TBI model on μ-calpain knockout mice showed that although m-calpain was activated in both wild type (WT) and KO mice, the proportion of cell death in μ-calpain KO mice was significantly higher than that of WT, suggesting that the activation of μ-calpain may have certain neuroprotective effects (Wang et al., 2018b). Other studies have also shown that expression of endogenous calpain inhibitor calpastatin to reduce the activation of calpain failed to prevent neuronal apoptosis (Schoch et al., 2013). On the other side, MAPK/ERK signaling pathway involves in the regulation of many proteins, of which calpain is only one of them. Functionally, MAPK signaling pathway is associated with apoptosis, differentiation, migration, proliferation, et cetera (Anderson and Tolkovsky, 1999; Ortega and Alcántara, 2010; Huo et al., 2015; Sun et al., 2015). In CNS, activation of the MAPK/ERK signaling pathway can help the cell survive by promoting neuron anti-oxidation and anti-glutamic toxicity (Zhao et al., 2013; Ortuño-Sahagún et al., 2014), while inhibition of MAPK/ERK signaling pathway induces apoptosis and inhibits cell proliferation (Roy et al., 2010; Anne et al., 2013). Thus, simple inhibition of MAPK/ERK pathway by using their inhibitors such as SL-327 or PD98059 may cause broad effect in the central nervous system. Taken together, suppressing of m-calpain, rather than inhibition of upstream MAPK/ERK activation or broad calpain inhibition, during seizure induction might be a suitable target for future anti-epileptic drug development.

**Figure 6:**
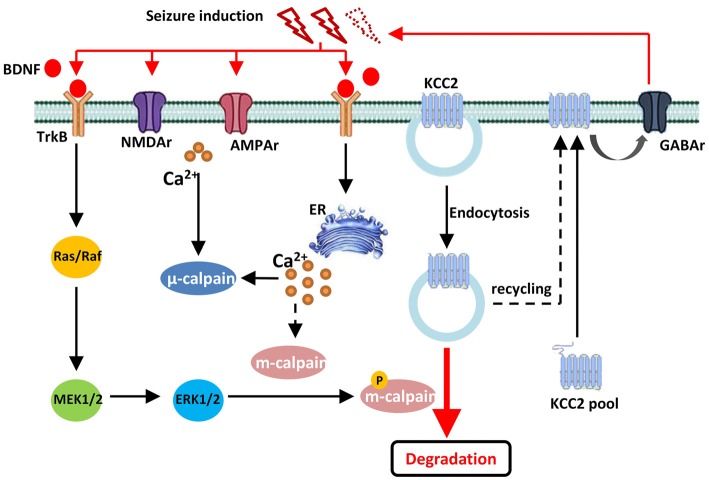
Drawing diagram showing the cellular mechanism of m-calpain phosphorylation involvement in seizure onset induced KCC2 down regulation. Both endocytosis and TrkB-MAPK/ERK–m-calpain pathways regulated KCC2 level in neurons.

In conclusion, our current study discovered that, during convulsant stimulation, m-calpain, but not μ-calpain, was over activated, which was partly regulated by calcium independent MAPK/ERK signaling pathway. The enhanced m-calpain activation, in turn, down regulated KCC2 expression, and hence facilitated seizure induction (Figure 6). Our results suggest, in conjunction with many other facts, that m-calpain might be a suitable target for anti-epileptic drug development.

## Author Contributions

LW is the main writer of this manuscript, he carried out *in vivo* recording, WB and IP experiments, and helped with the experiments design. LR contributed to experiments design and analysis of EEG data. LC carried out the immunostaining part. GW contributed to *in vitro* experiments and result analysis. XL assisted in the manuscript writing. BW helped with the WB experiments and YW guided the design and implementation of this research.

## Conflict of Interest Statement

The authors declare that the research was conducted in the absence of any commercial or financial relationships that could be construed as a potential conflict of interest.

## References

[B1] Anderson C. N. Tolkovsky A. M. (1999). A role for MAPK/ERK in sympathetic neuron survival: protection against a p53-dependent, JNK-independent induction of apoptosis by cytosine arabinoside. J. Neurosci. 19, 664–673. 10.1523/JNEUROSCI.19-02-00664.1999 9880587 PMC6782192

[B2] Anne S. L. Govek E. E. Ayrault O. Kim J. H. Zhu X. Murphy D. A.. (2013). WNT3 inhibits cerebellar granule neuron progenitor proliferation and medulloblastoma formation via MAPK activation. PLoS One 8:e81769. 10.1371/journal.pone.0081769 24303070 PMC3841149

[B3] Araujo I. M. Gil J. M. Carreira B. P. Mohapel P. Petersen A. Pinheiro P. S.. (2008). Calpain activation is involved in early caspase-independent neurodegeneration in the hippocampus following status epilepticus. J. Neurochem. 105, 666–676. 10.1111/j.1471-4159.2007.05181.x 18088374

[B4] Baek H. Yi M. H. Pandit S. Park J. B. Kwon H. H. Zhang E.. (2016). Altered expression of KCC2 in GABAergic interneuron contributes prenatal stress-induced epileptic spasms in infant rat. Neurochem. Int. 97, 57–64. 10.1016/j.neuint.2016.05.006 27180051

[B5] Baudry M. Bi X. (2016). Calpain-1 and calpain-2: the yin and yang of synaptic plasticity and neurodegeneration. Trends Neurosci. 39, 235–245. 10.1016/j.tins.2016.01.007 26874794 PMC4818674

[B6] Carr K. D. de Vaca S. C. Sun Y. Chau L. S. Pan Y. Dela Cruz J. (2009). Effects of the MEK inhibitor, SL-327, on rewarding, motor- and cellular-activating effects of D-amphetamine and SKF-82958 and their augmentation by food restriction in rat. Psychopharmacology 201, 495–506. 10.1007/s00213-008-1313-6 18766328 PMC2803695

[B7] Chamma I. Chevy Q. Poncer J. C. Lévi S. (2012). Role of the neuronal K-Cl co-transporter KCC2 in inhibitory and excitatory neurotransmission. Front. Cell. Neurosci. 6:5. 10.3389/fncel.2012.00005 22363264 PMC3282916

[B8] Chamma I. Heubl M. Chevy Q. Renner M. Moutkine I. Eugene E.. (2013). Activity-dependent regulation of the K/Cl transporter KCC2 membrane diffusion, clustering, and function in hippocampal neurons. J. Neurosci. 33, 15488–15503. 10.1523/JNEUROSCI.5889-12.2013 24068817 PMC6618451

[B9] Chen H. Libertini S. J. Wang Y. Kung H. J. Ghosh P. Mudryj M. (2010). ERK regulates calpain 2-induced androgen receptor proteolysis in CWR22 relapsed prostate tumor cell lines. J. Biol. Chem. 285, 2368–2374. 10.1074/jbc.M109.049379 19946123 PMC2807295

[B11] Chen L. Y. Rex C. S. Casale M. S. Gall C. M. Lynch G. (2007). Changes in synaptic morphology accompany actin signaling during LTP. J. Neurosci. 27, 5363–5372. 10.1523/JNEUROSCI.0164-07.2007 17507558 PMC6672340

[B10] Chen L. Wan L. Wu Z. Ren W. Huang Y. Qian B.. (2017). KCC2 downregulation facilitates epileptic seizures. Sci. Rep. 7:156. 10.1038/s41598-017-00196-7 28279020 PMC5427808

[B12] Eftekhari S. Mehrabi S. Soleimani M. Hassanzadeh G. Shahrokhi A. Mostafavi H.. (2014). BDNF modifies hippocampal KCC2 and NKCC1 expression in a temporal lobe epilepsy model. Acta Neurobiol. Exp. 74, 276–287. 25231847 10.55782/ane-2014-1993

[B13] Enomoto S. Shimizu K. Nibuya M. Suzuki E. Nagata K. Kondo T. (2017). Activated brain-derived neurotrophic factor/TrkB signaling in rat dorsal and ventral hippocampi following 10-day electroconvulsive seizure treatment. Neurosci. Lett. 660, 45–50. 10.1016/j.neulet.2017.09.011 28890399

[B14] Etehadi Moghadam S. Azami Tameh A. Vahidinia Z. Atlasi M. A. Hassani Bafrani H. Naderian H. (2018). Neuroprotective effects of oxytocin hormone after an experimental stroke model and the possible role of calpain-1. J. Stroke Cerebrovasc. Dis. 27, 724–732. 10.1016/j.jstrokecerebrovasdis.2017.10.020 29249590

[B15] Gellerman D. M. Bi X. Baudry M. (1997). NMDA receptor-mediated regulation of AMPA receptor properties in organotypic hippocampal slice cultures. J. Neurochem. 69, 131–136. 10.1046/j.1471-4159.1997.69010131.x 9202303

[B16] Glading A. Bodnar R. J. Reynolds I. J. Shiraha H. Satish L. Potter D. A.. (2004). Epidermal growth factor activates m-calpain (calpain II), at least in part, by extracellular signal-regulated kinase-mediated phosphorylation. Mol. Cell. Biol. 24, 2499–2512. 10.1128/mcb.24.6.2499-2512.2004 14993287 PMC355832

[B17] Glass J. D. Culver D. G. Levey A. I. Nash N. R. (2002). Very early activation of m-calpain in peripheral nerve during Wallerian degeneration. J. Neurol. Sci. 196, 9–20. 10.1016/s0022-510x(02)00013-8 11959150

[B18] Goll D. E. Thompson V. F. Li H. Wei W. Cong J. (2003). The calpain system. Physiol. Rev. 83, 731–801. 10.1152/physrev.00029.2002 12843408

[B19] González M. I. (2016). Regulation of the cell surface expression of chloride transporters during epileptogenesis. Neurosci. Lett. 628, 213–218. 10.1016/j.neulet.2016.06.042 27345384 PMC4969124

[B20] Griscavage J. M. Wilk S. Ignarro L. J. (1996). Inhibitors of the proteasome pathway interfere with induction of nitric oxide synthase in macrophages by blocking activation of transcription factor NF-kappa B. Proc. Natl. Acad. Sci. U S A 93, 3308–3312. 10.1073/pnas.93.8.3308 8622934 PMC39603

[B21] Hao F. Jia L. H. Li X. W. Zhang Y. R. Liu X. W. (2016). Garcinol upregulates GABAA and GAD65 expression, modulates BDNF-TrkB pathway to reduce seizures in pentylenetetrazole (PTZ)-induced epilepsy. Med. Sci. Monit. 22, 4415–4425. 10.12659/msm.897579 27855137 PMC5117238

[B22] Hosseini-Zare M. S. Salehi F. Seyedi S. Y. Azami K. Ghadiri T. Mobasseri M.. (2011). Effects of pentoxifylline and H-89 on epileptogenic activity of bucladesine in pentylenetetrazol-treated mice. Eur. J. Pharmacol. 670, 464–470. 10.1016/j.ejphar.2011.09.026 21946102

[B23] Huang X. McMahon J. Yang J. Shin D. Huang Y. (2012). Rapamycin down-regulates KCC2 expression and increases seizure susceptibility to convulsants in immature rats. Neuroscience 219, 33–47. 10.1016/j.neuroscience.2012.05.003 22613737 PMC3402618

[B24] Hübner C. A. (2014). The KCl-cotransporter KCC2 linked to epilepsy. EMBO Rep. 15, 732–733. 10.15252/embr.201439039 24928907 PMC4196972

[B25] Huo Y. N. Chen W. Zheng X. X. (2015). ROS, MAPK/ERK and PKC play distinct roles in EGF-stimulated human corneal cell proliferation and migration. Cell. Mol. Biol. 61, 6–11. 26567598

[B26] Kahle K. T. Khanna A. R. Duan J. Staley K. J. Delpire E. Poduri A. (2016). The KCC2 cotransporter and human epilepsy: getting excited about inhibition. Neuroscientist 22, 555–562. 10.1177/1073858416645087 27130838

[B27] Kahle K. T. Merner N. D. Friedel P. Silayeva L. Liang B. Khanna A.. (2014). Genetically encoded impairment of neuronal KCC2 cotransporter function in human idiopathic generalized epilepsy. EMBO Rep. 15, 766–774. 10.15252/embr.201438840 24928908 PMC4196980

[B28] Kahle K. T. Staley K. J. Nahed B. V. Gamba G. Hebert S. C. Lifton R. P.. (2008). Roles of the cation-chloride cotransporters in neurological disease. Nat. Clin. Pract. Neurol. 4, 490–503. 10.1038/ncpneuro0883 18769373

[B29] Karlócai M. R. Wittner L. Tóth K. Maglóczky Z. Katarova Z. Rásonyi G.. (2016). Enhanced expression of potassium-chloride cotransporter KCC2 in human temporal lobe epilepsy. Brain Struct. Funct. 221, 3601–3615. 10.1007/s00429-015-1122-8 26427846

[B30] Kong S. Qian B. Liu J. Fan M. Chen G. Wang Y. (2010). Cyclothiazide induces seizure behavior in freely moving rats. Brain Res. 1355, 207–213. 10.1016/j.brainres.2010.07.088 20678492 PMC2947190

[B31] Kupina N. C. Nath R. Bernath E. E. Inoue J. Mitsuyoshi A. Yuen P. W.. (2001). The novel calpain inhibitor SJA6017 improves functional outcome after delayed administration in a mouse model of diffuse brain injury. J. Neurotrauma 18, 1229–1240. 10.1089/089771501317095269 11721741

[B32] Lam P. M. Carlsen J. González M. I. (2017). A calpain inhibitor ameliorates seizure burden in an experimental model of temporal lobe epilepsy. Neurobiol. Dis. 102, 1–10. 10.1016/j.nbd.2017.02.003 28237317 PMC5640433

[B33] Lee H. H. Deeb T. Z. Walker J. A. Davies P. A. Moss S. J. (2011). NMDA receptor activity downregulates KCC2 resulting in depolarizing GABAA receptor-mediated currents. Nat. Neurosci. 14, 736–743. 10.3410/f.13360061.14730195 21532577 PMC3102766

[B34] Lee H. H. Walker J. A. Williams J. R. Goodier R. J. Payne J. A. Moss S. J. (2007). Direct protein kinase C-dependent phosphorylation regulates the cell surface stability and activity of the potassium chloride cotransporter KCC2. J. Biol. Chem. 282, 29777–29784. 10.1074/jbc.M705053200 17693402

[B35] Li W. Yu J. Liu Y. Huang X. Abumaria N. Zhu Y.. (2013). Elevation of brain magnesium prevents and reverses cognitive deficits and synaptic loss in Alzheimer’s disease mouse model. J. Neurosci. 33, 8423–8441. 10.1523/JNEUROSCI.4610-12.2013 23658180 PMC6619649

[B36] Li X. Zhou J. Chen Z. Chen S. Zhu F. Zhou L. (2008). Long-term expressional changes of Na^+^ -K^+^ -Cl^−^ co-transporter 1 (NKCC1) and K^+^ -Cl^−^ co-transporter 2 (KCC2) in CA1 region of hippocampus following lithium-pilocarpine induced status epilepticus (PISE). Brain Res. 1221, 141–146. 10.1016/j.brainres.2008.04.047 18550034

[B38] Liu X. Liu J. Liu J. Liu X. L. Jin L. Y. Fan W.. (2013). BDNF-TrkB signaling pathway is involved in pentylenetetrazole-evoked progression of epileptiform activity in hippocampal neurons in anesthetized rats. Neurosci. Bull. 29, 565–575. 10.1007/s12264-013-1326-y 23550026 PMC5561949

[B37] Liu J. Liu M. C. Wang K. K. (2008). Calpain in the CNS: from synaptic function to neurotoxicity. Sci. Signal. 1:re1. 10.1126/stke.114re1 18398107

[B39] Liu Y. Wang Y. Zhu G. Sun J. Bi X. Baudry M. (2016). A calpain-2 selective inhibitor enhances learning and memory by prolonging ERK activation. Neuropharmacology 105, 471–477. 10.1016/j.neuropharm.2016.02.022 26907807 PMC4873344

[B40] Loscher W. Puskarjov M. Kaila K. (2013). Cation-chloride cotransporters NKCC1 and KCC2 as potential targets for novel antiepileptic and antiepileptogenic treatments. Neuropharmacology 69, 62–74. 10.1016/j.neuropharm.2012.05.045 22705273

[B41] Mahadevan V. Woodin M. A. (2016). Regulation of neuronal chloride homeostasis by neuromodulators. J. Physiol. 594, 2593–2605. 10.1113/JP271593 26876607 PMC4865579

[B42] Maravall M. Mainen Z. F. Sabatini B. L. Svoboda K. (2000). Estimating intracellular calcium concentrations and buffering without wavelength ratioing. Biophys. J. 78, 2655–2667. 10.1016/s0006-3495(00)76809-3 10777761 PMC1300854

[B43] Medina I. Friedel P. Rivera C. Kahle K. T. Kourdougli N. Uvarov P.. (2014). Current view on the functional regulation of the neuronal K^+^-Cl^−^ cotransporter KCC2. Front. Cell. Neurosci. 8:27. 10.3389/fncel.2014.00027 24567703 PMC3915100

[B44] Mocchetti I. Bachis A. (2004). Brain-derived neurotrophic factor activation of TrkB protects neurons from HIV-1/gp120-induced cell death. Crit. Rev. Neurobiol. 16, 51–57. 10.1615/critrevneurobiol.v16.i12.50 15581399

[B45] Munakata M. Watanabe M. Otsuki T. Nakama H. Arima K. Itoh M.. (2007). Altered distribution of KCC2 in cortical dysplasia in patients with intractable epilepsy. Epilepsia 48, 837–844. 10.1111/j.1528-1167.2006.00954.x 17284302

[B46] Nam H. Y. Na E. J. Lee E. Kwon Y. Kim H. J. (2017). Antiepileptic and neuroprotective effects of oleamide in rat striatum on kainate-induced behavioral seizure and excitotoxic damage via calpain inhibition. Front. Pharmacol. 8:817. 10.3389/fphar.2017.00817 29209207 PMC5702338

[B47] Numakawa T. Suzuki S. Kumamaru E. Adachi N. Richards M. Kunugi H. (2010). BDNF function and intracellular signaling in neurons. Histol. Histopathol. 25, 237–258. 10.14670/HH-25.237 20017110

[B48] Ono Y. Saido T. C. Sorimachi H. (2016). Calpain research for drug discovery: challenges and potential. Nat. Rev. Drug Discov. 15, 854–876. 10.1038/nrd.2016.212 27833121

[B49] Ortega J. A. Alcántara S. (2010). BDNF/MAPK/ERK-induced BMP7 expression in the developing cerebral cortex induces premature radial glia differentiation and impairs neuronal migration. Cereb. Cortex 20, 2132–2144. 10.1093/cercor/bhp275 20038543

[B50] Ortuño-Sahagún D. González R. M. Verdaguer E. Huerta V. C. Torres-Mendoza B. M. Lemus L.. (2014). Glutamate excitotoxicity activates the MAPK/ERK signaling pathway and induces the survival of rat hippocampal neurons *in vivo*. J. Mol. Neurosci. 52, 366–377. 10.1007/s12031-013-0157-7 24190281

[B51] Papp A. Fehér O. Erdélyi L. (1987). The ionic mechanism of the pentylenetetrazol convulsions. Acta Biol. Hung. 38, 349–361. 3503442

[B52] Puskarjov M. Ahmad F. Kaila K. Blaesse P. (2012). Activity-dependent cleavage of the K-Cl cotransporter KCC2 mediated by calcium-activated protease calpain. J. Neurosci. 32, 11356–11364. 10.1523/JNEUROSCI.6265-11.2012 22895718 PMC6621186

[B53] Puskarjov M. Ahmad F. Khirug S. Sivakumaran S. Kaila K. Blaesse P. (2015). BDNF is required for seizure-induced but not developmental up-regulation of KCC2 in the neonatal hippocampus. Neuropharmacology 88, 103–109. 10.1016/j.neuropharm.2014.09.005 25229715

[B54] Qian B. Sun Y. Wu Z. Wan L. Chen L. Kong S.. (2011). Epileptiform response of CA1 neurones to convulsant stimulation by cyclothiazide, kainic acid and pentylenetetrazol in anaesthetized rats. Seizure 20, 312–319. 10.1016/j.seizure.2010.12.016 21269843

[B55] Rivera C. Li H. Thomas-Crusells J. Lahtinen H. Viitanen T. Nanobashvili A.. (2002). BDNF-induced TrkB activation down-regulates the K^+^-Cl^−^ cotransporter KCC2 and impairs neuronal Cl^−^ extrusion. J. Cell Biol. 159, 747–752. 10.1083/jcb.200209011 12473684 PMC2173387

[B56] Rosenberger G. Gal A. Kutsche K. (2005). αPIX associates with calpain 4, the small subunit of calpain and has a dual role in integrin-mediated cell spreading. J. Biol. Chem. 280, 6879–6889. 10.1074/jbc.M412119200 15611136

[B57] Roy S. K. Srivastava R. K. Shankar S. (2010). Inhibition of PI3K/AKT and MAPK/ERK pathways causes activation of FOXO transcription factor, leading to cell cycle arrest and apoptosis in pancreatic cancer. J. Mol. Signal. 5:10. 10.1186/1750-2187-5-10 20642839 PMC2915986

[B58] Schoch K. M. von Reyn C. R. Bian J. Telling G. C. Meaney D. F. Saatman K. E. (2013). Brain injury-induced proteolysis is reduced in a novel calpastatin-overexpressing transgenic mouse. J. Neurochem. 125, 909–920. 10.1111/jnc.12144 23305291 PMC3676438

[B59] Sierra-Paredes G. Cornes J. M. Sierra-Marcuno G. (1999). Calpain inhibitor I retards seizure offset in the hippocampus of freely moving rats. Neurosci. Lett. 263, 165–168. 10.1016/s0304-3940(99)00136-6 10213161

[B60] Silayeva L. Deeb T. Z. Hines R. M. Kelley M. R. Munoz M. B. Lee H. H.. (2015). KCC2 activity is critical in limiting the onset and severity of status epilepticus. Proc. Natl. Acad. Sci. U S A 112, 3523–3528. 10.1073/pnas.1415126112 25733865 PMC4371976

[B61] Sorimachi H. Hata S. Ono Y. (2011a). Calpain chronicle—an enzyme family under multidisciplinary characterization. Proc. Jpn. Acad. Ser. B Phys. Biol. Sci. 87, 287–327. 10.2183/pjab.87.287 21670566 PMC3153876

[B62] Sorimachi H. Hata S. Ono Y. (2011b). Impact of genetic insights into calpain biology. J. Biochem. 150, 23–37. 10.1093/jb/mvr070 21610046

[B63] Sun Y. Liu W. Z. Liu T. Feng X. Yang N. Zhou H. F. (2015). Signaling pathway of MAPK/ERK in cell proliferation, differentiation, migration, senescence and apoptosis. J. Recept. Signal Transduct. Res. 35, 600–604. 10.3109/10799893.2015.1030412 26096166

[B64] Wang Y. Bi X. Baudry M. (2018a). Calpain-2 as a therapeutic target for acute neuronal injury. Expert Opin. Ther. Targets 22, 19–29. 10.1080/14728222.2018.1409723 29168923 PMC6211856

[B65] Wang Y. Liu Y. Lopez D. Lee M. Dayal S. Hurtado A.. (2018b). Protection against TBI-induced neuronal death with post-treatment with a selective calpain-2 inhibitor in mice. J. Neurotrauma 35, 105–117. 10.1089/neu.2017.5024 28594313 PMC5757088

[B66] Wang Y. Lopez D. Davey P. G. Cameron D. J. Nguyen K. Tran J.. (2016). Calpain-1 and calpain-2 play opposite roles in retinal ganglion cell degeneration induced by retinal ischemia/reperfusion injury. Neurobiol. Dis. 93, 121–128. 10.1016/j.nbd.2016.05.007 27185592

[B67] Wang Y. Qi J. S. Kong S. Sun Y. Fan J. Jiang M.. (2009). BDNF-TrkB signaling pathway mediates the induction of epileptiform activity induced by a convulsant drug cyclothiazide. Neuropharmacology 57, 49–59. 10.1016/j.neuropharm.2009.04.007 19393251 PMC2733837

[B68] Wu H. Che X. Tang J. Ma F. Pan K. Zhao M.. (2016). The K^+^-Cl^−^ cotransporter KCC2 and chloride homeostasis: potential therapeutic target in acute central nervous system injury. Mol. Neurobiol. 53, 2141–2151. 10.1007/s12035-015-9162-x 25941074

[B69] Xu J. Kurup P. Zhang Y. Goebel-Goody S. M. Wu P. H. Hawasli A. H.. (2009). Extrasynaptic NMDA receptors couple preferentially to excitotoxicity via calpain-mediated cleavage of STEP. J. Neurosci. 29, 9330–9343. 10.1523/JNEUROSCI.2212-09.2009 19625523 PMC2737362

[B70] Yin Y. Wang Y. Gao D. Ye J. Wang X. Fang L.. (2016). Accumulation of human full-length tau induces degradation of nicotinic acetylcholine receptor α4 via activating calpain-2. Sci. Rep. 6:27283. 10.1038/srep27283 27277673 PMC4899694

[B71] Zadran S. Jourdi H. Rostamiani K. Qin Q. Bi X. Baudry M. (2010). Brain-derived neurotrophic factor and epidermal growth factor activate neuronal m-calpain via mitogen-activated protein kinase-dependent phosphorylation. J. Neurosci. 30, 1086–1095. 10.1523/JNEUROSCI.5120-09.2010 20089917 PMC2820881

[B72] Zanardelli S. Christodoulou N. Skourides P. A. (2013). Calpain2 protease: a new member of the Wnt/Ca^2+^ pathway modulating convergent extension movements in Xenopus. Dev. Biol. 384, 83–100. 10.1016/j.ydbio.2013.09.017 24076278

[B73] Zhao B. Wong A. Y. Murshid A. Bowie D. Presley J. F. Bedford F. K. (2008). Identification of a novel di-leucine motif mediating K^+^/Cl^−^ cotransporter KCC2 constitutive endocytosis. Cell. Signal. 20, 1769–1779. 10.1016/j.cellsig.2008.06.011 18625303

[B74] Zhao L. Wang J. L. Wang Y. R. Fa X. Z. (2013). Apigenin attenuates copper-mediated β-amyloid neurotoxicity through antioxidation, mitochondrion protection and MAPK signal inactivation in an AD cell model. Brain Res. 1492, 33–45. 10.1016/j.brainres.2012.11.019 23178511

[B75] Zhou H. Y. Chen S. R. Byun H. S. Chen H. Li L. Han H. D.. (2012). N-methyl-D-aspartate receptor- and calpain-mediated proteolytic cleavage of K^+^-Cl^−^ cotransporter-2 impairs spinal chloride homeostasis in neuropathic pain. J. Biol. Chem. 287, 33853–33864. 10.1074/jbc.M112.395830 22854961 PMC3460480

[B76] Zimmerman U. J. Schlaepfer W. W. (1984). Calcium-activated neutral protease (CANP) in brain and other tissues. Prog. Neurobiol. 23, 63–78. 10.1016/0301-0082(84)90012-1 6097938

