# Supplementary material for: M-Calpain Activation Facilitates Seizure Induced KCC2 Down Regulation
Source: Front Mol Neurosci. 2018 Aug 21;11:287. doi: 10.3389/fnmol.2018.00287 (PMC6110871; doi:10.3389/fnmol.2018.00287)
Supplement: Supplementary file 1 [file Data_Sheet_1.PDF]

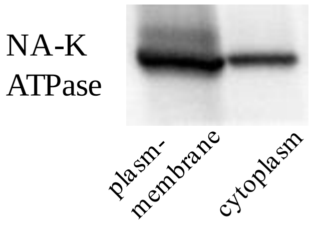

Figure S1. Plasm membrane and cytoplasm Na^+/K^+ ATPase blotting. Naïve hippocampus were extracted by using Membrane Protein Extraction Kit mentioned in “Material and Methods” for cytoplasm and membrane sample preparation. Samples were loaded onto the gel and run under the same antibody concentration, the same exposure time, and the same sample loading amount, and then visualized by Na^+/K^+ ATPase antibody.
